# Distinct microbiome profiles on vaginally inserted polypropylene midurethral mesh slings compared to vaginal, urinary, and skin microbiomes

**DOI:** 10.1128/aem.02463-24

**Published:** 2025-06-23

**Authors:** Nazish Abbas, Thomas Willmott, Paul M. Campbell, Gurdeep Singh, Maya Basu, Fiona Reid, Andrew J. McBain

**Affiliations:** 1 Division of Developmental Biology & Medicine, School of Medical Sciences, Faculty of Biology, Medicine and Health, University of Manchester 12203, Manchester, United Kingdom; 2 Warrell Unit, Saint Mary's Hospital, Manchester University NHS Foundation Trust 5293, Manchester, United Kingdom; 3 Manchester Academic Health Science Centre 158986, Manchester, United Kingdom; 4 Division of Pharmacy and Optometry, School of Health Sciences, Faculty of Biology, Medicine and Health, University of Manchester 12203, Manchester, United Kingdom; 5 Lydia Becker Institute of Immunology and Inflammation, Manchester Academic Health Science Centre, Faculty of Biology, Medicine and Health, University of Manchester 12203, Manchester, United Kingdom; Centers for Disease Control and Prevention, Atlanta, Georgia, USA

**Keywords:** mesh, microbiome, incontinence, midurethral slings

## Abstract

Midurethral slings are widely used in the treatment of stress urinary incontinence in women. However, little is known about the microbiomes that develop on these implants, their relationship to the urinary and vaginal microbiomes, or their potential role in mesh-related complications. In this study, we characterized the microbiomes of explanted midurethral slings and examined associations with clinical complications. Seventy-four women provided a total of 397 samples, including explanted mesh, urine, and swabs from the vagina and groin or suprapubic skin. Participants were categorized into clinical groups: chronic pain, vaginal mesh exposure, lower urinary tract perforation, or recurrent incontinence (control group). Samples underwent 16S rRNA gene sequencing. The mesh microbiome was dominated by Firmicutes, Proteobacteria, and Actinobacteria, with *Enterococcus* particularly abundant. Microbial diversity was significantly higher in mesh samples compared to vaginal and skin swabs, but not urine. The mesh microbiome was compositionally distinct from the urinary, vaginal, and skin microbiomes, potentially reflecting vaginal microbiome alterations due to urinary incontinence at the time of implantation. Differences in microbial diversity in mesh and skin samples among women with pain suggest a possible microbial contribution to mesh complications. These findings demonstrate the presence of distinct, site-specific microbial communities on explanted midurethral slings, with potential implications for understanding mesh-related complications.

**IMPORTANCE:**

Stress urinary incontinence commonly affects women, and effective treatment is essential. Midurethral mesh slings have provided effective relief; however, long-term complications such as chronic pain, vaginal mesh exposure, and lower urinary tract perforation have emerged. The pathophysiology of these complications is not well understood but is thought to involve a heightened inflammatory response to mesh implants. The local microbiome may contribute to this inflammation. We have shown that the mesh samples harbored a distinct microbiome and that differences in microbial composition may be associated with mesh complications. Understanding the role of specific bacteria in modulating host responses may offer new insights into the pathogenesis of mesh complications and inform future clinical approaches.

## INTRODUCTION

Midurethral mesh slings (MUS), also called tapes, were regarded as the gold standard treatment for stress urinary incontinence (SUI) (1). However, long-term complications have come to light which have led to a pause in the use of vaginally implanted mesh in several countries, including the UK (2). These long-term complications include chronic pain, vaginal mesh exposure (the presence of uncovered mesh within the vagina), and lower urinary tract perforation (mesh within the bladder or urethra) (3). Many women who have suffered mesh complications feel that they have come to significant harm (4–6). The pathophysiology of these complications has not been fully defined, but studies examining explanted mesh have described persistent inflammation at the site of complications and that bacteria may colonize mesh (7–12).

Changes in the human microbiome have been associated with various diseases (13, 14). In women, the vaginal microbiome is relatively stable in health, with a dominance of lactobacilli, although shifts throughout life stages have been reported (15). The vaginal microbiome may also be altered by a variety of factors that include smoking (16), hygiene (17), preterm labor (18), gynaecological cancers (19), and bacterial vaginosis (20)—an imbalance in the composition of the vaginal microbiota. Changes in the urinary microbiome have also been associated with urgency incontinence (21, 22) and bladder cancers (23, 24).

To the best of our knowledge, only two studies have been published to date investigating the relationship of the microbiome with mesh complications, and these have focused only on the vaginal microbiome (25, 26). One further study has assessed the bacteria present on the mesh using culture techniques (27). Given the limited number of studies that have examined the mesh microbiome, there is a need for further investigation of the composition and origins via characterization of the mesh and local microbiomes surrounding the surgical site. We have, therefore, characterized the mesh microbiomes and their relationship with various complications, as well as with the surrounding local microbiomes of the vagina, urine, and skin at the mesh arm exit sites.

## MATERIALS AND METHODS

### Study design

Eligible women were recruited (see the supplemental material for complete inclusion/exclusion criteria), with all participating women providing informed written consent.

### Study participants

Participants were recruited consecutively and allocated to two groups: case and control (see the supplemental material for details). Women entered the case group if they had a MUS with a long-term complication and were undergoing surgery to excise their MUS. Women entered the control group if they had an MUS without long-term complications. Some of the women who entered the control group were identified at clinic attendance and not undergoing mesh excision surgery; these women did not donate mesh samples for research. The reason for the addition of these women was to include a control group, as it is not usual practice to excise mesh without complications, due to the additional risks of surgery. Women with more than one MUS or clinical signs of mesh infection were excluded from this study. Information on antibiotic, probiotic, and vaginal oestrogen usage was collected at the time of recruitment (Table S1). A sample size of convenience was used to recruit maximal numbers of women undergoing MUS excision within one unit over a 2-year period.

### Sample collection, storage, and standardization

Swabs were collected as E-swabs (Sterilab, United Kingdom). Samples were stored frozen at −80°C and thawed at 37°C prior to genomic extraction. A total of 3 mL of urine was stored frozen at −80°C in a sterile pot for each woman. Once ready for microbial genomic extraction, samples were thawed at 37°C and then 1 mL of urine was centrifuged at 13,300 × *g* for 10 min at 24°C to form a pellet. The supernatant was discarded, and the pellet was resuspended in 100 µL of the remaining urine. Mesh was stored frozen at −80°C in 3 mL phosphate-buffered saline (PBS). Mesh was allowed to thaw at 37°C for 1 h and then weighed. As samples were of varying sizes, each sample was standardized to 0.1 g by cutting with sterile scissors, except where the sample was too small. If the total mesh sample was lighter than 0.1 g, the entire sample was used, and the lower weight recorded.

### Pilot study samples

Prior to being granted ethical approval for the collection of swabs and urine for this study, women undergoing midurethral mesh sling excision were invited to donate their mesh samples to the Manchester University NHS Foundation Trust Biobank. These samples were referred to as “pilot study samples,” as these are not paired with swabs or urine. These mesh samples were included in the current study and were collected, processed, and treated in the same way as all other mesh samples.

### DNA extraction, amplification, and sequencing

DNA was extracted using the DNeasy PowerSoil Pro Kit (Qiagen, Manchester, UK) and used for 16S rRNA sequencing analysis (Illumina system) using adapted 515F and 806R primers (28). All DNA samples were extracted, amplified, purified, and sequenced in the same batch. Raw sequence data were imported into the Quantitative Insights into Microbial Ecology (QIIME) version 2 (2020.2) (29, 30) and data subsequently processed in R version 3.6.2 (31) and expressed as bacterial relative abundances (32–38). Statistical investigation of differential analysis of count data was accomplished with DESeq2 which corrects the *P*-values for multiple testing using the Benjamini and Hochberg method by default (39). Full details of bioinformatic analysis are outlined in the supplemental material. Throughout for all diversity analyses, *P* < 0.05 was considered significant.

## RESULTS

Across a 24-month period, 74 women were recruited and completed the study, totaling 397 samples analyzed (Fig. S1). The demographic details of pilot study participants are summarized in Table S1.

### Microbiome composition

Initially, all mesh samples (*n* = 180) were analyzed together. One sample did not generate reads, and this was removed from further analysis (Table S2). The phyla of the mesh microbiome were characterized by 51.8% Firmicutes, 21.2% Proteobacteria, 14.2% Actinobacteria, 5.1% Fusobacteria, and 4% Bacteroidetes. The Firmicutes phylum was dominated by *Enterococcus* which made up 24% of the mesh microbiome, followed by Lactobacillus which corresponded to 8.4% (Table S3).

No reads were obtained for three urine samples, and these were excluded from further analysis. The remaining urine samples (*n* = 51) were used to characterize the urinary microbiome. Firmicutes made up 51.2% of the relative abundance, Proteobacteria 26.1%, Actinobacteria 13.6%, Fusobacteria 3.7%, and Bacteroidetes 2.9% (Table S4). One vaginal swab sample was removed from analysis as it did not yield any reads, and all other samples were retained for analysis (*n* = 55). The vaginal microbiome was 60.6% Firmicutes, 15.9% Proteobacteria, 15.3% Actinobacteria, and 5.2% Bacteroidetes (Table S5). One sample of skin swabs was removed as it did not yield any reads. The remaining skin swabs (*n* = 106) were used to characterize the skin microbiome from the mons pubis and groins. Firmicutes corresponded to 66.3% of the relative abundance at the phylum level, followed by 19.3% Actinobacteria, 11.6% Proteobacteria, 1.2% Fusobacteria, and 1.2% Bacteroidetes (Table S6).

### Comparing the mesh microbiome with the local microbiome

**Fig 1:**
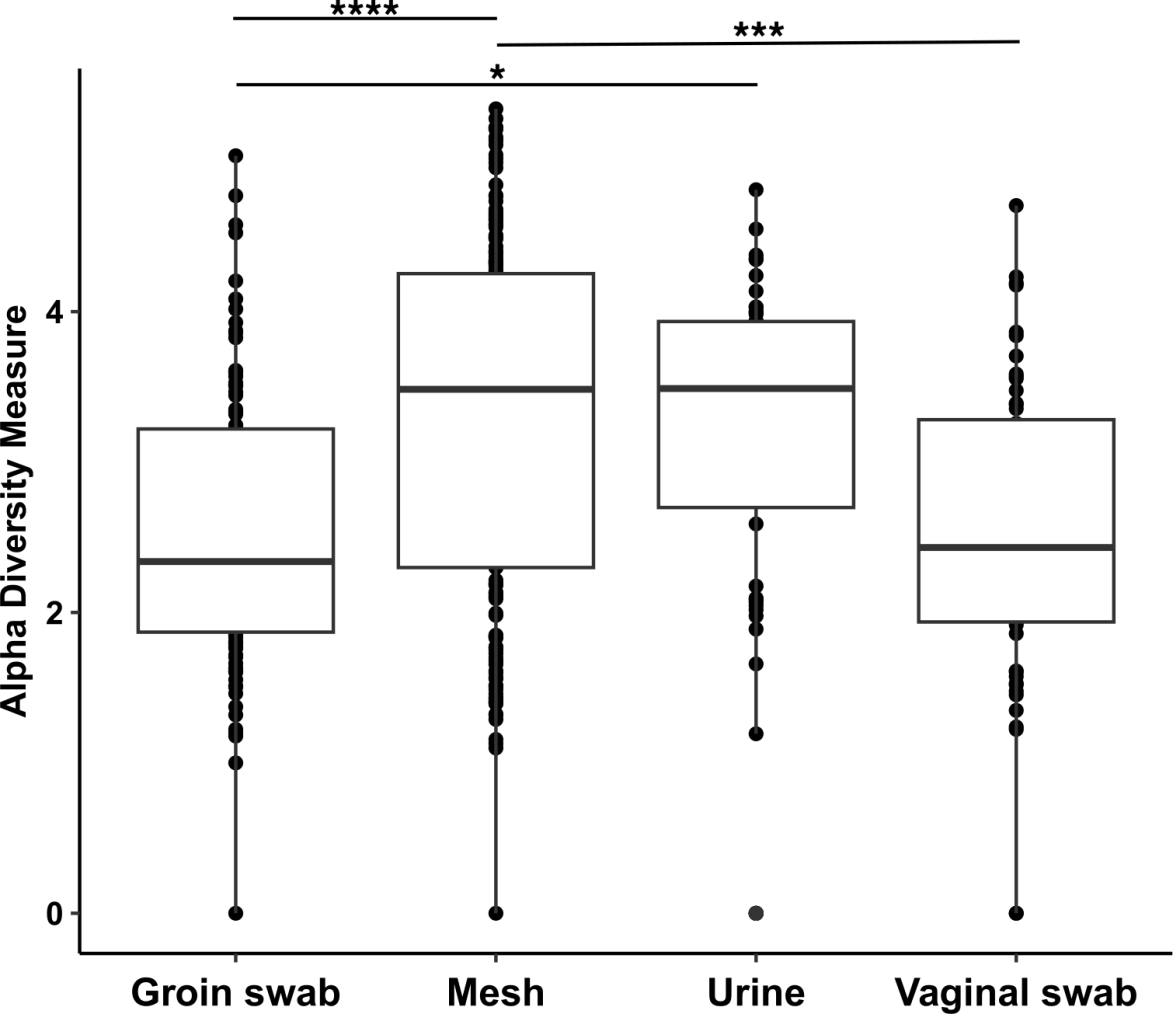
Shannon alpha diversity (richness) of microbiome samples taken from groin skin swabs, mesh, urine, and vaginal swabs. There was significantly higher alpha diversity in the mesh samples compared to the groin (*P* < 0.0001) and vaginal swabs (*P* < 0.001) and the urine compared to the groin skin swabs (*P* = 0.003) (Table 1) (*P < 0.05, **P < 0.01, **P < 0.001, ****P < 0.0001).

To determine which local site the mesh microbiome may have originated from, the diversity of mesh samples was compared to the urinary, vaginal, and skin microbiome. Alpha diversity refers to the species diversity (richness) within a functional community (40). Alpha diversity was compared between all groups using ANOVA, followed by post-hoc Tukey test. The alpha diversity of the mesh was significantly higher compared to the vagina (*P* = 0.001) and groin skin (*P* = 0.0001), but not significantly different to urine (*P* = 0.81) (Fig. 1 and Table 1).

**TABLE 1:** Post-hoc Tukey of ANOVA comparing alpha diversity between all samples

	95% Confidence interval	*P* value
Mesh-Groin	0.4–1.1	<0.0001
Urine-Groin	0.2–1.1	0.003
Vagina-Groin	−1	1
Urine-Mesh	−0.9	0.81
Vagina-Mesh	−0.9	<0.001
Vagina-Mesh	−1	0.016

**Fig 2:**
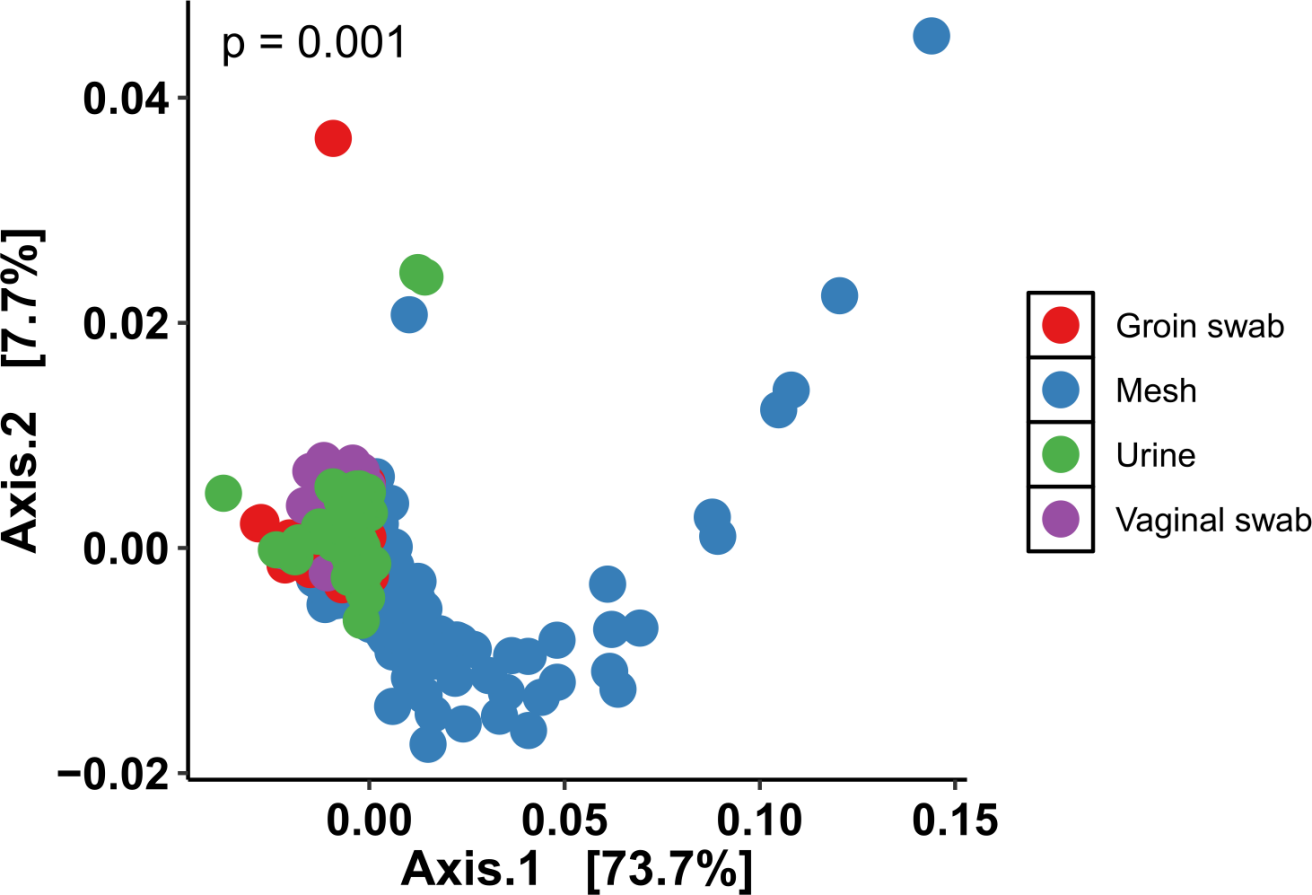
Principal components analysis plotting beta diversity (*P* = 0.001) between microbiome samples taken from groin swabs, mesh, urine, and vaginal swabs (weighted UniFrac analysis; ADONIS analysis of variance using distance matrices).

Beta diversity is an ecological measure of similarity between multiple communities, comparing changes in community composition between categorical different samples (40). Here, beta diversity was calculated using weighted Unifrac, which is a quantitative measure of beta diversity which detects changes in how many sequences from each lineage are present (41). Microbiome data are presented in a principal coordinate analysis (PCoA), in which points together represent microbial communities that are more similar in sequence composition (42). The mesh microbiome was significantly different from the urinary, skin, and vaginal microbiomes (all *P* < 0.001) (Fig. 2).

**Fig 3:**
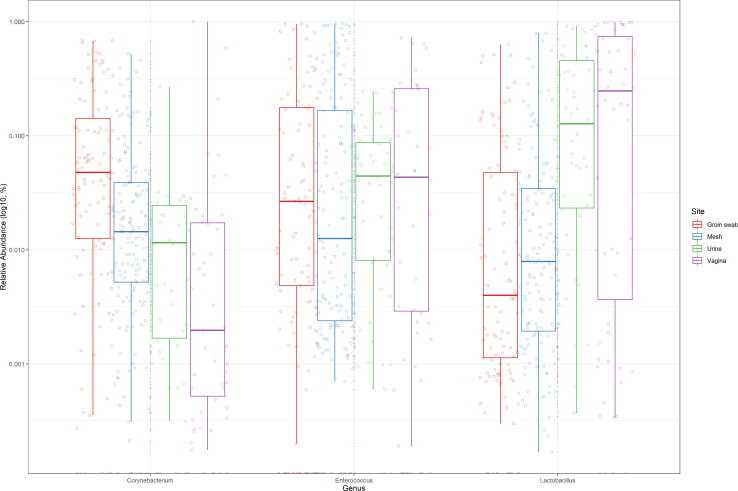
Relative abundance of the genera *Enterococcus*, *Corynebacterium*, and Lactobacillus by sample type (see Fig. 4). Data analyzed using post-hoc Tukey analysis and presented on a log Y scale. There were significantly higher relative abundances of *Enterococcus* in the mesh samples compared to the urine (*P* < 0.05). Groin swabs from the skin had the highest abundance of *Corynebacterium* compared to all other sites (*P* < 0.0001). Vaginal swabs and urine had higher Lactobacillus abundances compared to the mesh and groin swabs (*P* < 0.0001).

When grouping by site microbiome, the relative abundance of the three largest detected genera, *Enterococcus*, *Corynebacterium,* and *Lactobacilli,* was compared (Fig. 3). There were significantly higher relative abundances of *Enterococcus* in the mesh samples compared to the urine (*P* < 0.05). Groin swabs from the skin had the highest abundance of *Corynebacterium* compared to all other sites (*P* < 0.0001). Vaginal swabs and urine had higher Lactobacillus abundances compared to the mesh and groin swabs (*P* < 0.0001).

**Fig 4:**
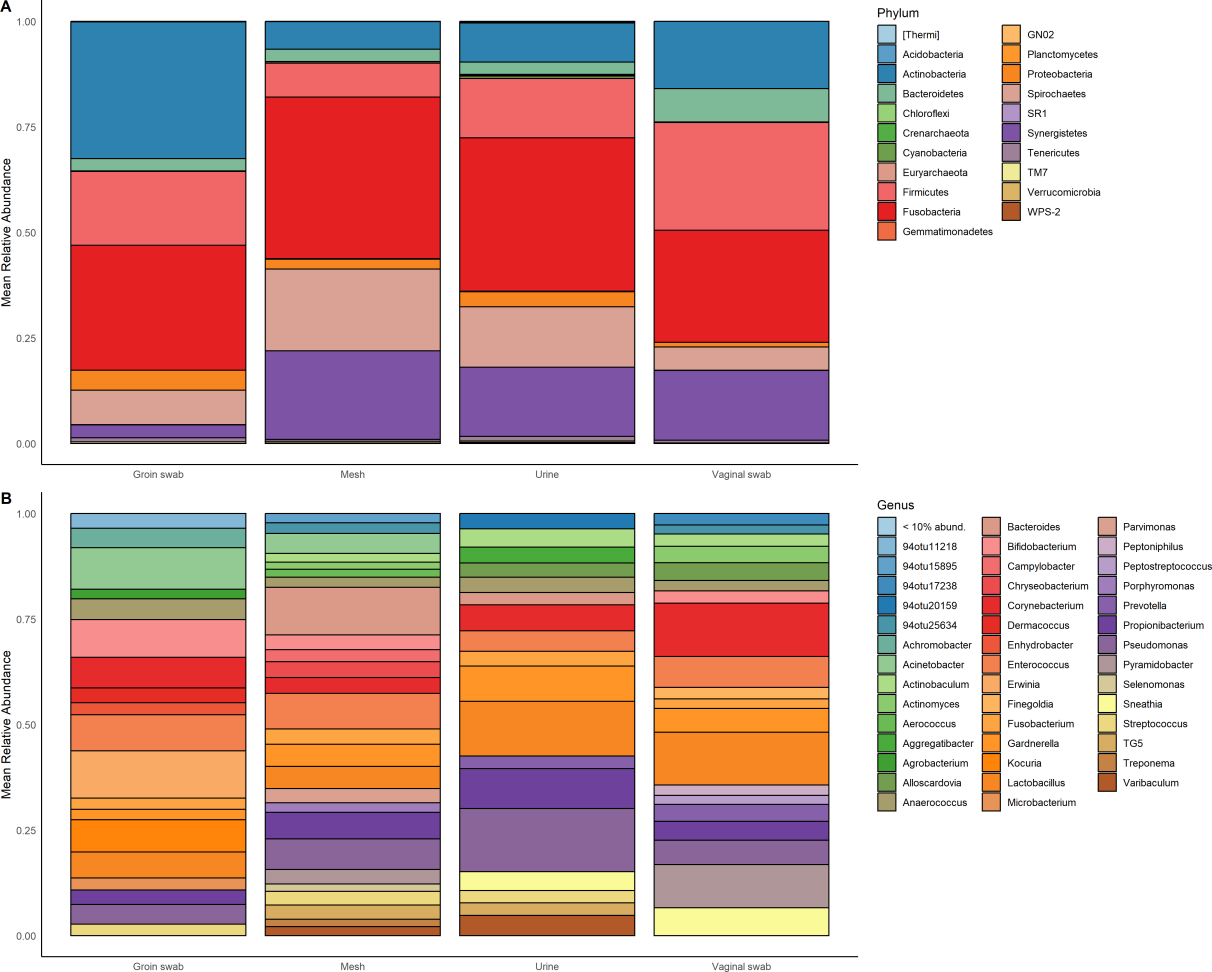
Microbiome profiles of groin swabs, MUS, urine, and vaginal mesh samples collected from women undergoing MUS excision surgery (*N* = 74). The stacked bar chart shows the mean proportion (relative abundance) at each sample site. Each taxon is represented by a different color and taxa found at relative abundance below 10% are grouped together. Relative abundances of bacterial taxa by sample site. Distribution of bacteria taxa is presented at the (**A**) phylum and (**B**) genus level.

**Fig 5:**
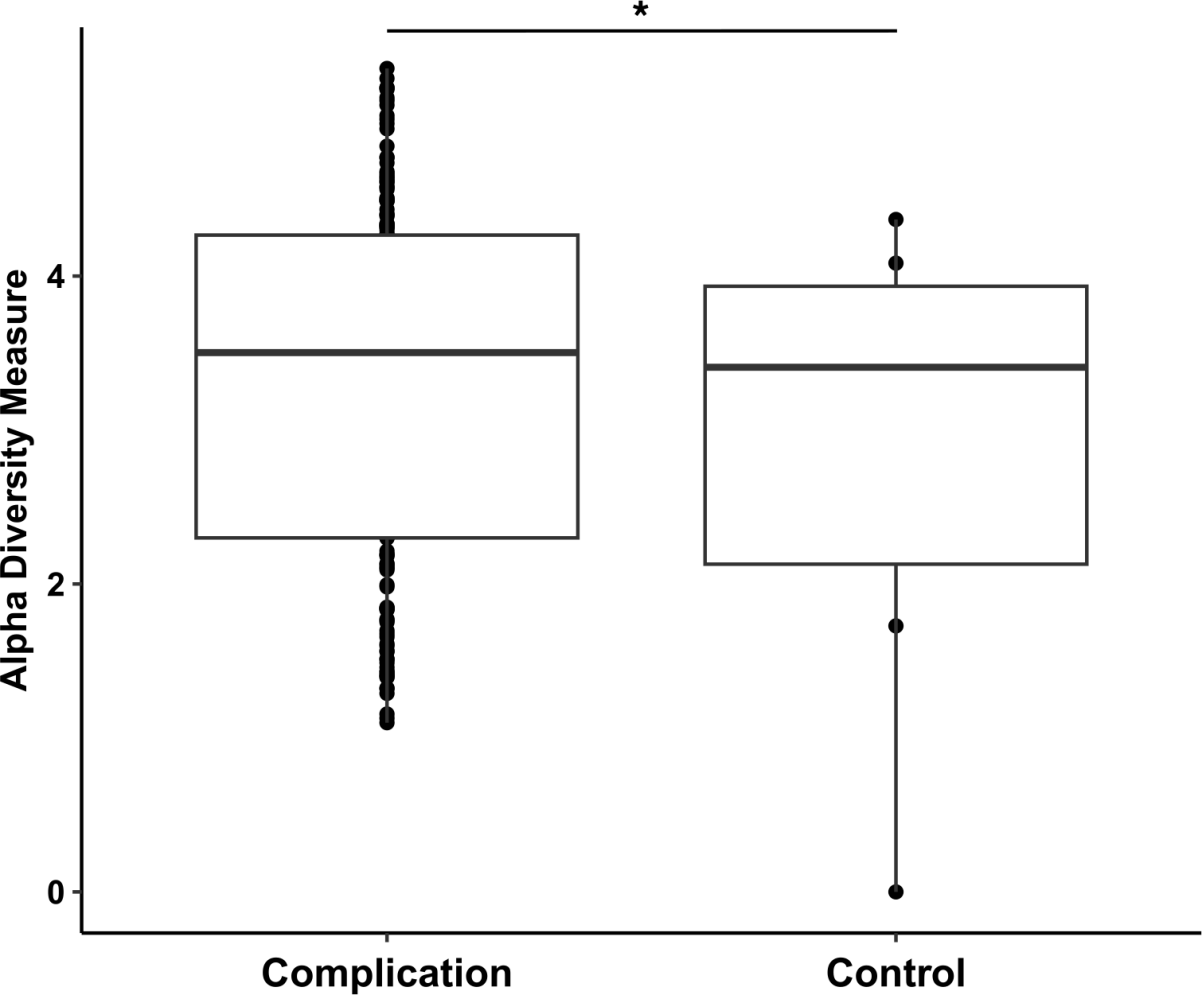
Shannon alpha diversity (richness) of microbiome samples taken from mesh, grouped into either complication (*n* = 30) and controls (*n* = 6) groups. No significant differences in alpha diversity of mesh samples by complication or control group (*P* > 0.05). (*P < 0.05, **P < 0.01, **P < 0.001, ****P < 0.0001).

**Fig 6:**
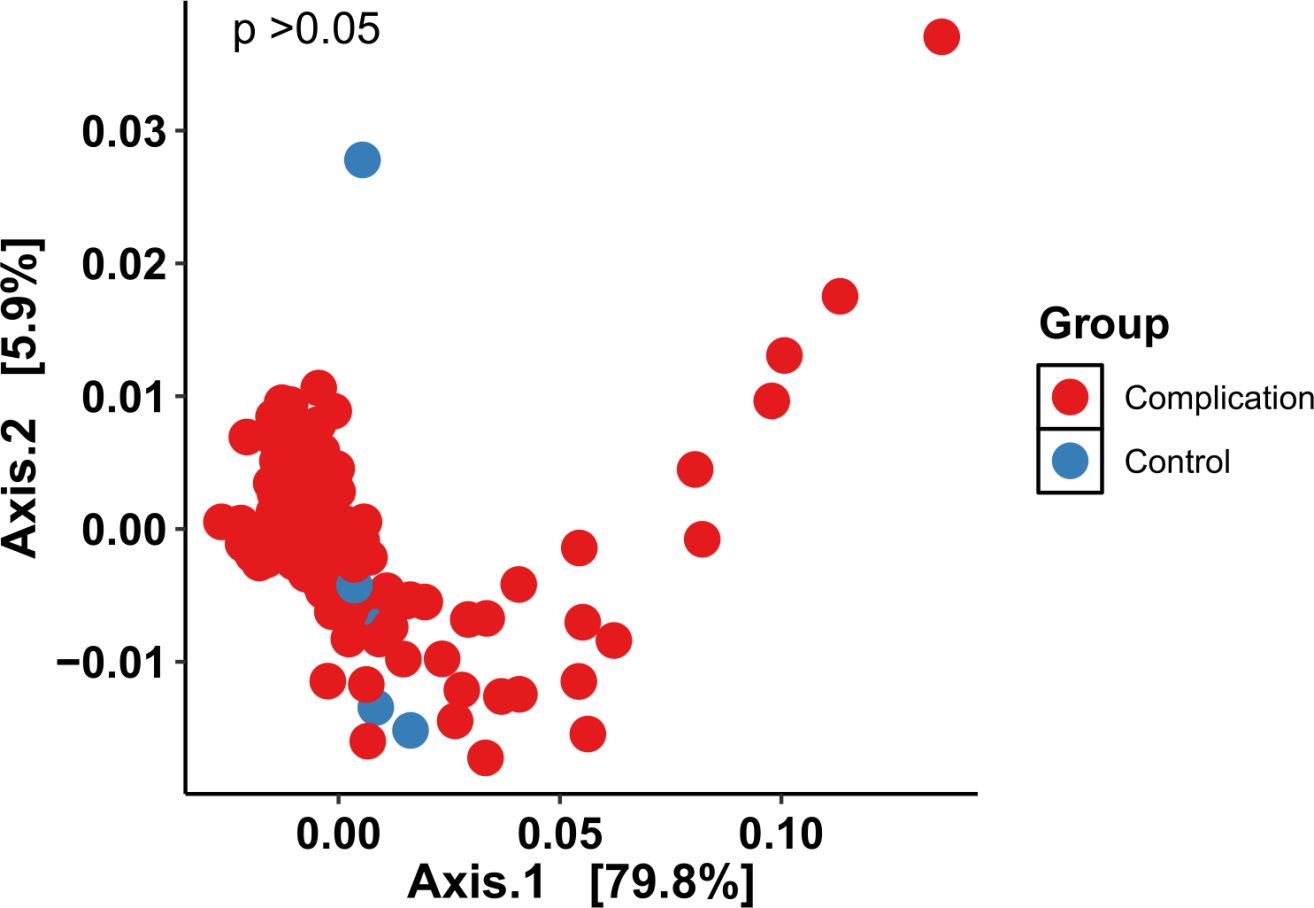
Principal component analysis to compare beta diversity of mesh samples by complication (*n* = 30) and control (*n* = 6) groups. No significant difference was found when comparing distribution by central tendency (*P* > 0.05).

Mesh samples were compared based on whether they corresponded with a complication (complication group: excision for pain, vaginal mesh exposure, or LUT perforation; *n* = 174, 69 women) or recurrent SUI surgery (control group, *n* = 6, 3 women). There was no significant difference in alpha diversity between these two groups (*P* > 0.05) (Fig. 5). Beta diversity was not significantly different between the two groups (*P* = 0.06) (Fig. 6).

Several genera were only present on the mesh and one other microbiome (Tables S2 to S5). These were *Propionibacterium*, *TG5*, and *Parvimonas* (urine), and *Finegoldia* (Vagina). Interestingly, *Campylobacter* was recorded on the mesh but on no other microbiome. Other site-specific genera included *Aggregatibacter*, *Prevotella*, *Actinobaculum*, *Varibaculum,* and *Porphyromonas* (Urine) and *Kocuria*, *Staphylococcus*, *Acintobacter*, *Neisseria,* and *Erwinia* (Skin).

### Mesh complications and the microbiome

**Fig 7:**
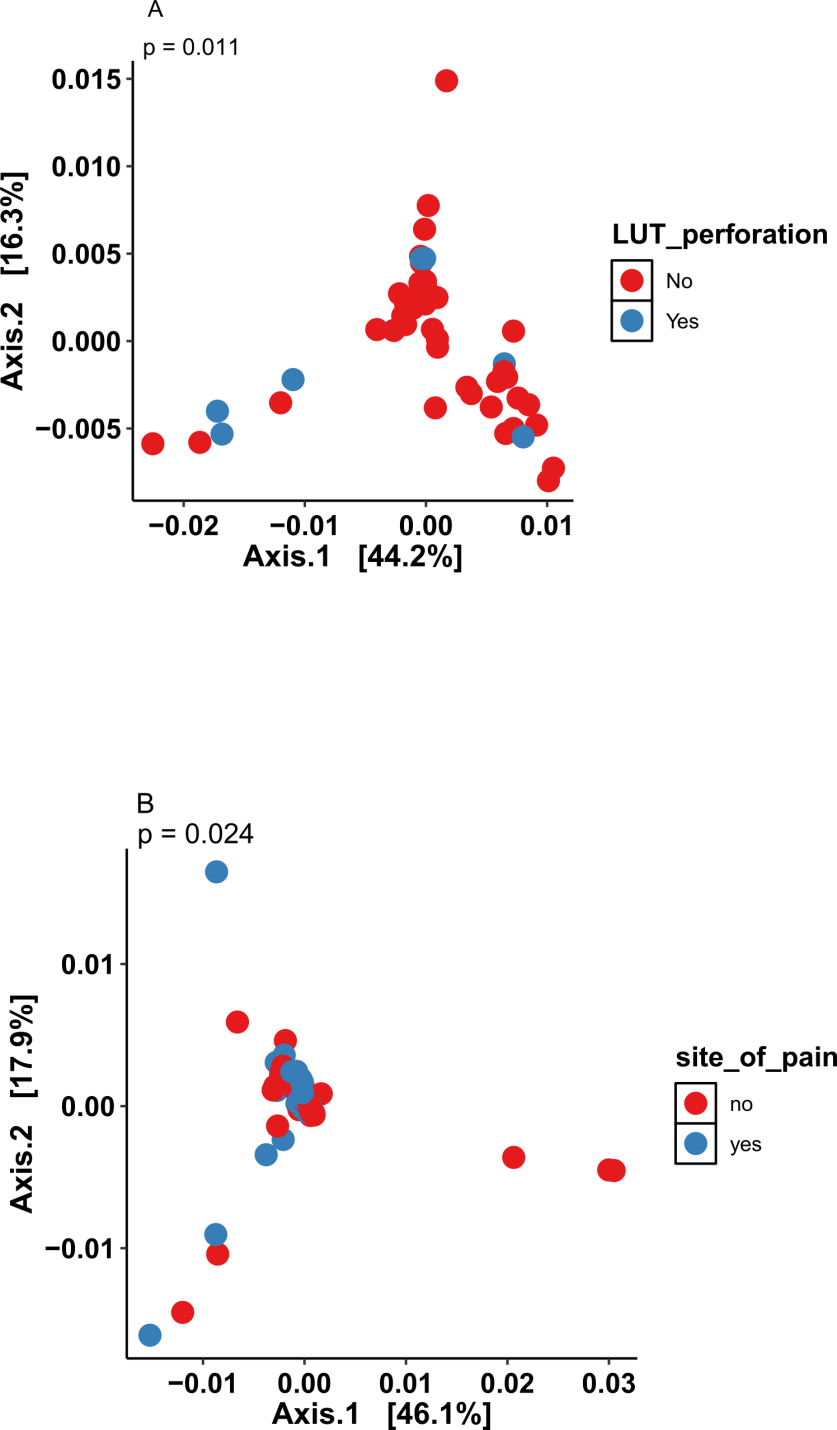
Principal component analysis comparing the beta diversity of the urinary microbiome of those with (**A**) LUT perforation (*P* = 0.011) and (**B**) the skin microbiome with pain (*P* = 0.024)—(Yes) to those without (No).

The urinary microbiome was then compared in those with LUT perforation (*n* = 7/51, 7 women) to those without LUT perforation. There was no significant difference in alpha diversity (*P* = 0.3817); however, there was a significant difference in beta diversity (*P* = 0.011) (Fig. 7A). Skin swabs were grouped into sites which corresponded with pain (*n* = 49) or not (*n* = 57). There were no significant differences in alpha diversity (*P* = 0.1365). However, beta diversity was significantly different between the two groups (*P* = 0.024) (Fig. 7B).

### Vaginal mesh exposure and the microbiome

**Fig 8:**
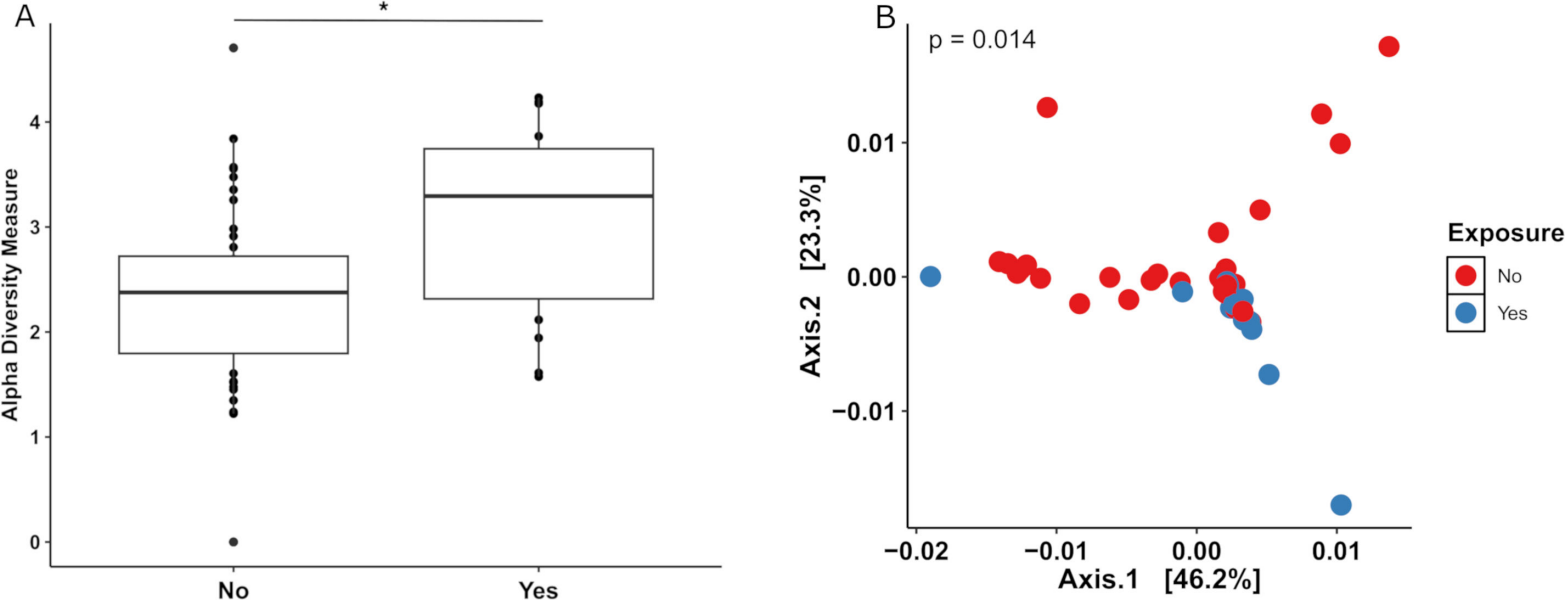
Comparison of diversity of the vaginal microbiome between women with (*n* = 16) and without (*n* = 39) vaginal mesh exposure. (**A**) Histogram comparing alpha diversity of the vaginal microbiome in those with vaginal mesh exposure (Yes) compared to those without (No). (**B**) Principal component analysis of the beta diversity of the vaginal microbiome, comparing those with vaginal mesh exposure (Yes) to those without (No). For both analyses, the two groups were significantly different, *P* < 0.05. (*P < 0.05, **P < 0.01, **P < 0.001, ****P < 0.0001).

Vaginal swabs were compared considering whether vaginal mesh exposure (*n* = 16/55) was present or not (*n* = 39/55). There was a greater alpha diversity associated with vaginal mesh exposure (*P* = 0.00632), Fig. 8A. Beta diversity was also significantly different between the two groups (*P* = 0.014), Fig. 8B. Fig. S2 represents significantly different OTUs, those above the red line were present in those with exposure, and those below in those without exposure. *Prevotella, Porphyromonas, Peptostreptococcus, Megasphaera,* and *Sneathia* were all more abundant in those with vaginal mesh exposure; lactobacilli were more common in those without mesh exposure.

## DISCUSSION

Marked differences were observed between the implanted mesh microbiome and the host urine, vaginal, and skin microbiomes. Although the midurethral mesh slings (MUS) are sterile when inserted, and patients undergo surgical site preparation to reduce the risk of infections, our data indicate that implanted MUS harbor bacteria, even when there is no evidence of frank infection. Bacterial DNA was detected throughout the mesh samples, not just restricted to the vaginal portion. As women with frank infection were excluded and bacterial DNA was detected on all mesh samples, the persistence of bacteria on polypropylene (PPM) without signs of clinical infection, including in the control group of women without mesh complications, is indicated.

There are currently few studies in the literature describing the microbiome in midurethral mesh slings. Previous studies assessing the link between the microbiome and mesh complications have focused on the vaginal microbiome (25, 26). The present study suggests that the vaginal microbiome cannot be used as a surrogate for the mesh microbiome. Furthermore, distinct vaginal microbiome compositions were detected in patients in which the mesh is exposed externally within the vagina, with significantly lower abundance of lactobacilli. It is not clear whether such differences between the vaginal microbiome are a result of mesh exposure. It would be possible to monitor this through a longitudinal study of women with characterization of the vaginal microbiome before mesh implantation, post-implantation, and long-term follow-up to detect those with vaginal mesh exposure. However, as this is a relatively uncommon complication, recruiting a sufficient number of participants who undergo long-term follow-up and remain in the study may prove logistically difficult.

Urinary tract perforation was associated with a significantly higher relative abundance of *Prevotella* in the urinary microbiome. *Prevotella* is principally an oral commensal, which has been previously detected in the urinary microbiome (22, 43). In the present study, the direction of the relationship between a higher relative abundance of *Prevotella* and LUT perforation is unknown; this study cannot deduce whether the presence of mesh in the LUT increases *Prevotella* or if a higher relative abundance of *Prevotella* in the urinary tract predisposes women to a LUT perforation. One study reported that women who benefited from MUS surgery, compared to those who did not, had higher abundances of *Prevotella* within the vaginal microbiome but no significant differences in the urinary microbiome (44).

Sites associated with pain had higher abundances of *Acinetobacter*, *Capnocytophaga*, *Fusobacterium,* and *Pseudomonas*. The development of pain after mesh implantation has been related to a heightened inflammatory response with excessive fibrosis and tissue remodeling (11, 45). The propensity of microorganisms to cause inflammation of host tissues depends on multiple factors, including their taxonomic identity and physiological traits, such as biofilm formation. It is, therefore, possible that mesh-related complications, such as pain, may be causally linked to the composition or physiology of the microbiota that colonize the mesh. This could help explain the broad interindividual variation observed clinically. Further investigation is needed to explore this possibility.

At the time of implantation, all women will have suffered from urinary incontinence. The leakage of urine may expose the vaginal microbiome to the urinary microbiome. It is possible that the vaginal microbiome more closely resembles the urinary microbiome when the MUS is implanted. This may, in part, explain the dissimilarities between the four sites sampled in this study. It is possible to see that urinary incontinence may lead to exposure of the vaginal microbiome to a different microbial community, and even directly change the vaginal environment, such as the pH and substrates available to metabolize, which would be beneficial to measure in future studies. Previous studies report that the vaginal and urinary microbiomes may be closely related (46). A longitudinal study of the vaginal and urinary microbiome in women undergoing MUS implantation reported that *Prevotella* abundance was associated with treatment failure, while lactobacilli dominance was associated with success, until age was controlled for (44). Therefore, further investigation of the relationship between the vaginal and urine microbiome and its association with MUS colonisation is warranted.

Previous studies provide evidence for bacterial colonisation of MUS; however, these studies, while highly informative, have been limited, to some extent, by their use of culture-based techniques (27, 47). Therefore, the full spectrum of bacterial diversity may have been underestimated. Some other studies of the mesh microbiome did not assess the mesh microbiome itself, being limited to the vaginal microbiome (25, 26). Further research aiming to look specifically at key organisms at the species level may help further our understanding of the role of lactobacilli or *Prevotella* in mesh complications and genitourinary health, especially considering that lactobacilli are regarded as a key player in the maintenance of health within the vaginal and urinary microbiomes.

Future research may involve characterizing the vaginal (48, 49) and mesh mycobiome and its association with complications; this would provide a more detailed analysis of the microbial community which exists on the mesh. Furthermore, research should aim to characterize the impact of the changing microbiome on the overall host health. This may require immunohistochemical analysis of stored samples and a comparison of this between complication groups and microbial communities. Here, we demonstrate that a unique microbiome is formed on inserted PPM meshes.

### Conclusion

Implanted mesh is sterile at the time of insertion, and women undergo routine surgical site preparation to reduce the risk of surgical site infection. Regardless, this study indicates that bacteria may colonize mesh and then persist. The study excluded women with frank infection, so the data generated provide evidence for the persistence of bacteria on PPM without signs of clinical infection, including in a control group of women without mesh complications. Mesh-associated communities were compositionally distinct from host local microbiomes, and bacterial DNA was detected throughout the whole length of the MUS, not just the vaginal portion. The differences in the diversity of mesh and skin microbiomes in women with and without pain may be indicative of a microbiome contribution to mesh complications which warrants further investigation.

## Data Availability

The data sets from this study are available in NCBI under BioProject accession number PRJNA1240690.

## SUPPLEMENTAL MATERIAL

Supplemental material aem.02463-24-s0001.docx Supplemental methods, Fig. S1 and S2, and Tables S1 to S6.

ASM does not own the copyrights to Supplemental Material that may be linked to, or accessed through, an article. The authors have granted ASM a non-exclusive, world-wide license to publish the Supplemental Material files. Please contact the corresponding author directly for reuse.
