# Supplementary material for: Distinct microbiome profiles on vaginally inserted polypropylene midurethral mesh slings compared to vaginal, urinary, and skin microbiomes
Source: Appl Environ Microbiol. 2025 Jun 23;91(7):e02463-24. doi: 10.1128/aem.02463-24 (PMC12285226; doi:10.1128/aem.02463-24)
Supplement: Supplemental material — Supplemental methods, Fig. S1 and S2, and Tables S1 to S6. [file aem.02463-24-s0001.docx]

**Materials and Methods**

**Study participants**

The two groups are summarised below.

1. Case:

Women undergoing mesh excision with one of the three complications: chronic pain attributed to mesh, vaginal mesh exposure, and lower urinary tract perforation of mesh.

2. Control

Women undergoing mesh excision without any of the above long-term complications. Women with an MUS without any long-term complication and attending a urogynaecology clinic for any other reason, e.g., recurrent stress incontinence or prolapse.

All women undergoing MUS excision surgery at Saint Mary’s Hospital, Manchester University NHS Foundation Trust, were considered for this study. Those who were undergoing mesh excision for the complications of chronic pain, lower urinary tract perforation or vaginal exposure were invited to join the case group, and those undergoing mesh excision as a concomitant procedure for repeat incontinence surgery were invited into the control group. At the outset of this study, we aimed to recruit 50 women into the study, and an additional 20 women who did not have MUS complications or recurrent stress incontinence who would be invited for a TLUS. This was a convenience sample, to allow recruitment within the timeframe proposed at the start of this study. Unfortunately, due to the Covid-19 pandemic, there was limited capacity for recruitment into the control group within the timeframe.

The following inclusion and exclusion criteria applied for all women:

Inclusion Criteria:

- Previous MUS procedure
- Over 18 years of age
- Able to give informed consent
- Able to complete questionnaires in English

Exclusion Criteria:

- Legally incapacitated for mental health or medical reasons preventing informed consent
- Under the age of 18 years
- Not able to understand written material in English

**DNA Extraction and Amplification**

After defrosting to room temperature and 30 seconds mixing with a benchtop vortex, 250μL of mesh/swab solution was extracted in the DNeasy PowerSoil Kit (Qiagen, Hilden, Germany) following the manufacturer’s instructions to isolate microbial DNA. Subsequent amplification was achieved through 16S rRNA gene PCR with Illumina (San Diego, USA) adapted 515F (5'NNNNNGTGCCAGCMGCCGCGGTAA3') and 806R (5'GGACTACHVGGGTWTCTAAT3') primers [1] and NEBNext^®^ High-Fidelity 2x PCR Master Mix (New England Biolabs, Ipswich, USA) at 98°C (2 minutes) followed by 25 cycles of 95°C (20 seconds), 62°C (15 seconds), 70°C, (30 seconds) and a final elongation step of 72°C (5 minutes). The amplified 16S rRNA gene was purified using a QIAquick purification kit (Qiagen, Hilden, Germany). Amplicons were confirmed via gel electrophoresis using 1% agarose gels.

**16S rRNA Gene Sequencing and Bioinformatics**

Amplified DNA was adjusted to 1ng/μL in a final volume of 20μL. Sequencing of 16S V4 amplicons was performed on the Illumina MiSeq platform (Illumina Inc, Cambridge, UK). Raw sequence data was imported into the quantitative insights into microbial ecology (QIIME) version 2 (2020.2) [2, 3]. Sequences were de-replicated, similarity clustered, analysed for chimeras, demultiplexed and quality filtered using the d2-demux plug-in followed by denoising with DADA2 (q2-dada2) [4]. Amplicon sequence variants (ASVs) were aligned via the q2-alignment plug-in and taxonomy assigned to ASVs using the q2-feature-classifier [5] against the Greengenes (v13.8) 97% ASV reference sequences [6] for the generation of BIOM tables comprising the sample metadata. Data was subsequently processed in R version 3.6.2 [7]. Data was imported using the qiime2R package [8], analysed using the Phyloseq package [9] and plots generated using the ggplot2 package [10]. Community richness (alpha diversity) was calculated using Shannon’s Richness (quantitative) and beta diversity (community dissimilarity) was calculated using weighted UniFrac (quantitative incorporating phylogenetic relationships). Statistical investigation of differential analysis of count data was accomplished with DESeq2 which corrects the p-values for multiple testing using the Benjamini and Hochberg method by default [11]. Data is presented as bacterial relative abundances, which is compositional data adding up to a sum constant, displaying the proportion of the taxa in the microbiome [12].

**Supplementary Figures and Tables**

**Figure S1.** Types of sample divided by case or control groups.


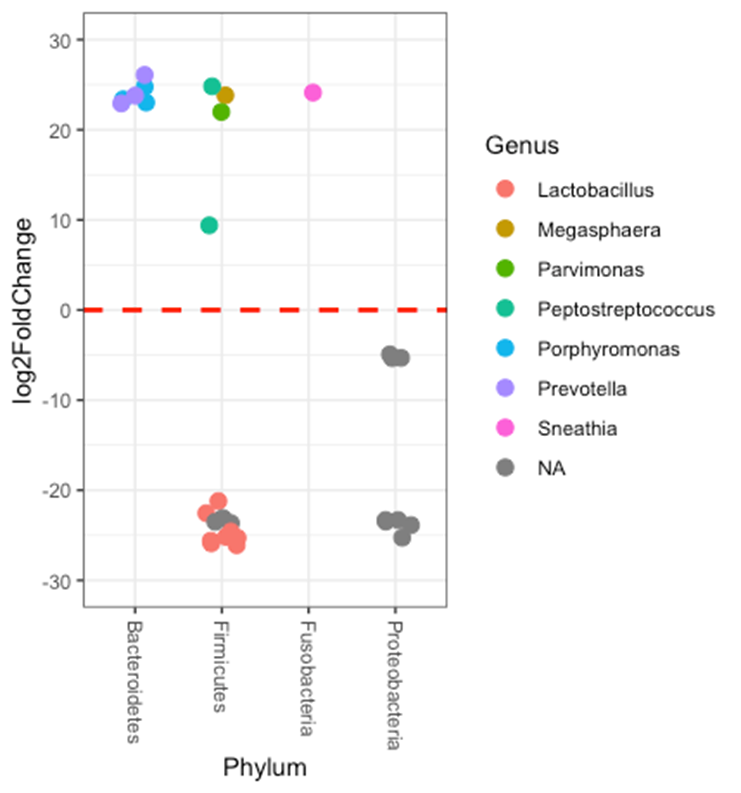


**Figure S2.** Significantly different OTUs in the vaginal microbiome, comparing those with vaginal mesh exposure (above the red line) to those without (below the red line) (P<0.05).

**Table S1.** Demographics of the study groups (n=57).

| Participant Demographic: |  |
| --- | --- |
| Age (Median)  (range)  [IQR] | 54  (37-73)  [10] |
| Body Mass Index (Median)  (range)  [IQR] | (31)  (19.7-38.7)  [8] |
| Smoking status | Yes = 7 (12%)  Ex-smoker = 8 (14%)  No = 42 (74%) |
| Diabetes | Yes = 2 (4%)  No = 55 (96%) |
| Autoimmune condition | Yes = 5 (9%)  No = 51 (89%)  Unreported = 1 (2%) |
| Oestrogen use | Yes = 7 (12%)  No = 50 (88%) |
| Probiotic use | Yes = 12 (21%)  No = 45 (79%) |
| Long term antibiotic use | Yes = 13 (23%)  No = 44 (77%) |
| MUS Demographic: |  |
| Years since insertion (Median)  (range)  [IQR] | (10)  (3-21)  [6] |
| Types of MUS | Obturator = 31 (42%)  Retropubic = 41 (55%)  Both = 1 (1%) |
| Complication | Control = 5 (7%)  Vaginal mesh exposure = 23 (31%)  Lower urinary perforation = 14 (19%)  Pain = 32 (43%) |
| Primary Indication for Mesh Excision: |  |
| Pain | 25 (43.9%) |
| Vaginal mesh exposure | 18 (31.6%) |
| Lower urinary tract perforation | 9 (15.8%) |
| Urethra | 8 (14%) |
| Bladder | 1 (1.8%) |
| Control | 5 (8.8%) |
| Self-Reported Symptoms: |  |
| Pain | Yes = 50 (88%)  No = 7 (12%) |
| Dyspareunia | Yes = 34 (60%)  No = 23 (40%) |
| Vaginal bleeding | Yes = 19 (33%)  No = 38 (67%) |
| Vaginal discharge | Yes = 14 (25%)  No = 43 (75%) |
| Recurrent UTIs | Yes = 28 (49%)  No = 29 (51%) |
| Leakage related to urgency | Yes = 39 (68%)  No = 18 (32%) |
| Leakage related to movement | Yes = 41 (72%)  No = 16 (28%) |
| Difficulty emptying bladder | Yes = 29 (51%)  No = 28 (49%) |

**Table S2.** NGS summary of mesh and host microbiomes.

| **Sample** | | **Reads** | | | | |
| --- | --- | --- | --- | --- | --- | --- |
| **Type** | **Number** | **Min** | **Max** | **Mean** | **Median** | **SD** |
| **Mesh** | 179 | 1 | 115209 | 44995.4022 | 41718 | 20025.8726 |
| **Vaginal Swabs** | 56 | 5 | 126342 | 45879.125 | 38295 | 25500.8318 |
| **Groin swabs** | 107 | 4 | 211961 | 49542.486 | 41184 | 36316.951 |
| **Urine** | 54 | 1 | 186273 | 40414.037 | 39351 | 26405.1 |

**Table S3.** Relative abundance of the mesh microbiome at the genus level.

| Phylum | Genus | Relative Abundance (%) |
| --- | --- | --- |
| Firmicutes | *Enterococcus* | 24.3 |
| Actinobacteria | *Corynebacterium* | 9.0 |
| Firmicutes | *Lactobacillus* | 8.4 |
| Fusobacteria | *Fusobacterium* | 6.3 |
| Proteobacteria | *Pseudomonas* | 6.1 |
| Firmicutes | *Streptococcus* | 3.5 |
| Actinobacteria | *Propionibacterium* | 3.3 |
| Actinobacteria | *Gardnerella* | 2.8 |
| Proteobacteria | *TG5* | 2.7 |
| Firmicutes | *Anaerococcus* | 2.2 |
| Firmicutes | *Parvimonas* | 2.0 |
| Bacteroidetes | *Prevotella* | 1.9 |
| Firmicutes | *Peptoniphilus* | 1.6 |
| Actinobacteria | *Actinomyces* | 1.6 |
| Firmicutes | *Finegoldia* | 1.4 |
| Proteobacteria | *Campylobacter* | 1.0 |

**Table S4.** Relative abundance of the urine microbiome at the genus level.

| Phylum | Genus | Relative Abundance (%) |
| --- | --- | --- |
| Firmicutes | *Lactobacillus* | 31.5 |
| Firmicutes | *Enterococcus* | 9.9 |
| Fusobacteria | *Fusobacterium* | 5.2 |
| Actinobacteria | *Gardnerella* | 4.7 |
| Proteobacteria | *Aggregatibacter* | 4.2 |
| Proteobacteria | *Pseudomonas* | 4.1 |
| Firmicutes | *Streptococcus* | 3.0 |
| Actinobacteria | *Corynebacterium* | 2.5 |
| Bacteroidetes | *Prevotella* | 2.2 |
| Proteobacteria | *TG5* | 2.0 |
| Actinobacteria | *Actinobaculum* | 1.9 |
| Firmicutes | *Parvimonas* | 1.9 |
| Actinobacteria | *Propionibacterium* | 1.8 |
| Firmicutes | *Anaerococcus* | 1.7 |
| Firmicutes | *Peptoniphilus* | 1.6 |
| Actinobacteria | *Actinomyces* | 1.3 |
| Actinobacteria | *Varibaculum* | 1.2 |
| Actinobacteria | *Alloscardovia* | 1.2 |
| Bacteroidetes | *Porphyromonas* | 1.0 |

**Table S5.** Relative abundance of the vagina microbiome at the genus level.

| Phylum | Genus | Relative Abundance (%) |
| --- | --- | --- |
| Firmicutes | *Lactobacillus* | 40.0 |
| Firmicutes | *Enterococcus* | 15.3 |
| Actinobacteria | *Gardnerella* | 8.4 |
| Actinobacteria | *Corynebacterium* | 7.9 |
| Bacteriodetes | *Prevotella* | 4.5 |
| Firmicutes | *Anaerococcus* | 1.8 |
| Firmicutes | *Finegoldia* | 1.8 |
| Proteobacteria | *Pseudomonas* | 1.4 |
| Firmicutes | *Peptoniphilus* | 1.4 |
| Fusobacteria | *Fusobacterium* | 1.4 |
| Actinobacteria | *Bifidobacterium* | 1.1 |
| Actinobacteria | *Alloscardovia* | 1.0 |

**Table S6.** Relative abundance of the skin microbiome at the genus level.

| Phylum | Genus | Relative Abundance (%) |
| --- | --- | --- |
| Actinobacteria | *Corynebacterium* | 30.6 |
| Firmicutes | *Enterococcus* | 20.2 |
| Firmicutes | *Lactobacillus* | 10.5 |
| Actinobacteria | *Kocuria* | 5.0 |
| Firmicutes | *Anaerococcus* | 3.0 |
| Fusobacteria | *Fusobacterium* | 2.7 |
| Firmicutes | *Streptococcus* | 2.6 |
| Firmicutes | *Staphylococcus* | 2.1 |
| Proteobacteria | *Acinetobacter* | 1.6 |
| Proteobacteria | *Pseudomonas* | 1.5 |
| Proteobacteria | *Neisseria* | 1.5 |
| Proteobacteria | *Erwinia* | 1.3 |
| Bacteriodetes | *Prevotella* | 1.0 |
| Actinobacteria | *Bifidobacterium* | 1.0 |

**References**

1. Caporaso, J.G., et al., *Ultra-high-throughput microbial community analysis on the Illumina HiSeq and MiSeq platforms.* Isme j, 2012. **6**(8): p. 1621-4.

2. Caporaso, J.G., et al., *QIIME allows analysis of high-throughput community sequencing data.* Nat Methods, 2010. **7**(5): p. 335-6.

3. Bolyen, E., et al., *Reproducible, interactive, scalable and extensible microbiome data science using QIIME 2.* Nat Biotechnol, 2019. **37**(8): p. 852-857.

4. Callahan, B.J., et al., *Bioconductor Workflow for Microbiome Data Analysis: from raw reads to community analyses.* F1000Res, 2016. **5**: p. 1492.

5. Bokulich, N.A., et al., *Optimizing taxonomic classification of marker-gene amplicon sequences with QIIME 2's q2-feature-classifier plugin.* Microbiome, 2018. **6**(1): p. 90.

6. McDonald, D., et al., *An improved Greengenes taxonomy with explicit ranks for ecological and evolutionary analyses of bacteria and archaea.* Isme j, 2012. **6**(3): p. 610-8.

7. RStudioTeam, *RStudio: Integrated Development for R*. 2020, RStudio.

8. Bisanz, J.E., *qiime2R: Importing QIIME2 artifacts and associated data into R sessions.* 2018. **0.99**.

9. McMurdie, P.J. and S. Holmes, *Phyloseq: a bioconductor package for handling and analysis of high-throughput phylogenetic sequence data.* Pac Symp Biocomput, 2012: p. 235-46.

10. Wickham, H., *ggplot2: Elegant Graphics for Data Analysis.* Springer-Verlag New York, 2016.

11. Love, M.I., W. Huber, and S. Anders, *Moderated estimation of fold change and dispersion for RNA-seq data with DESeq2.* Genome Biol, 2014. **15**(12): p. 550.

12. Galazzo, G., et al., *How to Count Our Microbes? The Effect of Different Quantitative Microbiome Profiling Approaches.* Front Cell Infect Microbiol, 2020. **10**: p. 403.
